# Supplementary material for: The interactome of intact mitochondria by cross-linking mass spectrometry provides evidence for coexisting respiratory supercomplexes
Source: Mol Cell Proteomics. 2017 Dec 8;17(2):216–32. doi: 10.1074/mcp.RA117.000470 (PMC5795388; doi:10.1074/mcp.RA117.000470)
Supplement: Supplemental Data [file supp_RA117.000470_133922_0_supp_23978_fzffwf.zip › spectra_annotation/mito_DR_spectra_annotation/347-1-4-1-5-1.pdf]

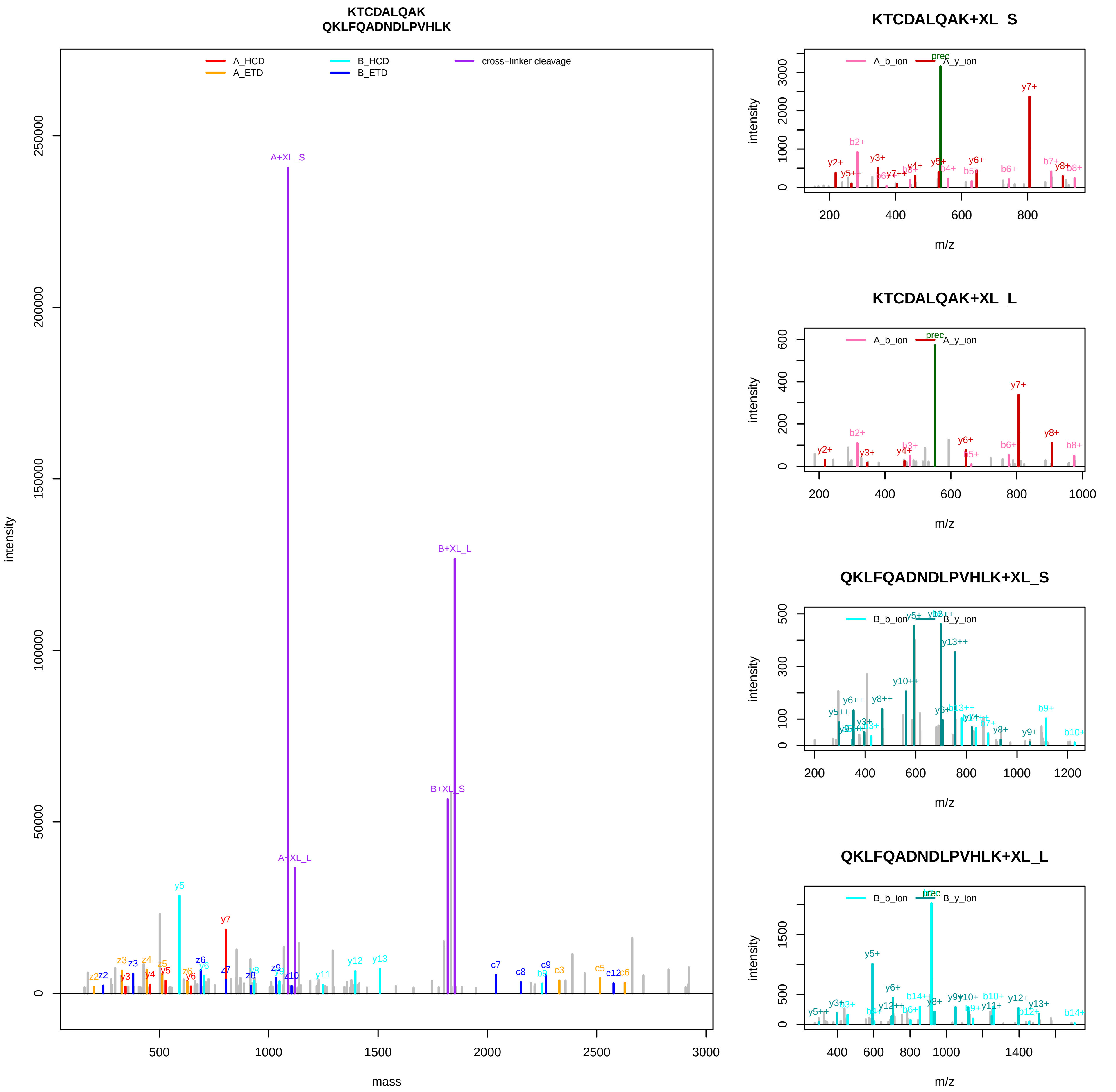

KTCDALQAK
QKLFQADNDLPVHLK
A_HCD
A_ETD
B_HCD
B_ETD
cross−linker cleavage
A+XL_S
A+XL_L
B+XL_L
B+XL_S
y5
y7
y12
y13
c7
c8
c9
c3
c5
c12
c6
intensity
mass
0
50000
100000
150000
200000
250000
500
1000
1500
2000
2500
3000
KTCDALQAK+XL_S
A_b_ion
A_y_ion
prec
y7+
b2+
y2+
y3+
y5++
y4+
y5+
y6+
b6+
b7+
y8+
b8+
m/z
intensity
KTCDALQAK+XL_L
A_b_ion
A_y_ion
prec
y7+
b2+
y2+
y3+
y4+
b3+
y6+
b6+
y8+
b8+
m/z
intensity
QKLFQADNDLPVHLK+XL_S
B_b_ion
B_y_ion
y5+
y12++
y13++
y10++
y6++
y8++
y5++
y6+
b13++
y7+
b7+
y8+
b9+
y9+
b10+
m/z
intensity
QKLFQADNDLPVHLK+XL_L
B_b_ion
B_y_ion
prec
y5+
y6+
b14++
y8+
y9+
y10+
b10+
y12+
y13+
y11+
b12+
b14+
y5++
y3+
b3+
y12++
m/z
intensity
